# Factors influencing the confidence and knowledge of nurses prescribing antiretroviral treatment in a rural and urban district in the Western Cape province

**DOI:** 10.4102/sajhivmed.v20i1.923

**Published:** 2019-07-02

**Authors:** Deborah J. Solomons, Anita S. van der Merwe, Tonya M. Esterhuizen, Talitha Crowley

**Affiliations:** 1 Department of Nursing and Midwifery, Faculty of Medicine and Health Sciences, Stellenbosch University, Cape Town, South Africa; 2 Division of Epidemiology and Biostatistics, Department of Global Health, Faculty of Medicine and Health Sciences, Stellenbosch University, Cape Town, South Africa

**Keywords:** HIV, NIMART, Primary Healthcare, Clinics, Nurses

## Abstract

**Background:**

Since the introduction of nurse-initiated and managed antiretroviral treatment (NIMART) in South Africa in 2010, initiation of antiretroviral therapy (ART) in primary care has become the responsibility of nurses. The continued success of this approach is dependent on factors such as adequate training and effective support systems.

**Objectives:**

This study aimed to investigate factors influencing the knowledge and confidence of professional nurses in managing patients living with human immunodeficiency virus (HIV) in primary healthcare settings in a rural and urban district in the Western Cape.

**Methods:**

A cross-sectional survey was conducted amongst 77 NIMART-trained nurses from 29 healthcare facilities to measure demographic details, influencing factors, HIV management confidence and HIV management knowledge.

**Results:**

The majority of participants had adequate HIV management knowledge and reported being very confident or expert in the HIV management skills or competencies. Participants trained recently on local guidelines (Practical Approach to Care Kit) (3 years ago or less) had significantly higher knowledge scores. Regular feedback about clinic and personal performance was associated with higher HIV management knowledge. Participants who received NIMART mentoring over a period of 2 weeks had a higher mean confidence score compared to other periods of mentoring. A higher caseload of patients living with HIV was also associated with higher knowledge and confidence.

**Conclusion:**

Training, mentorship and clinical practice experience are associated with knowledge and confidence. Recommendations include the strengthening of current training and mentoring and ensuring that NIMART-trained nurses are provided with regular updates and sufficient opportunities for clinical practice.

## Introduction and background

South Africa has the largest antiretroviral treatment programme in the world.^1^ Antiretroviral treatment guidelines are continuously revised, consequently increasing the threshold for antiretroviral therapy (ART) treatment. Because of persistent human resource constraints in South Africa, task shifting from doctors to nurses to prescribe ART became essential to ensure that more patients living with human immunodeficiency virus (HIV) are initiated on life-saving ART.^2^ Since the introduction of nurse-initiated and managed antiretroviral therapy (NIMART) in South Africa in 2010, there has been an increased demand for the training of professional nurses in HIV management in the primary healthcare (PHC) setting. The availability of NIMART-trained nurses in PHC clinics has improved the access of patients to HIV treatment.^3^

Although the shifting of tasks is a timely solution for human resource constraints, the continued success of the approach depends on factors such as adequate training and effective support systems.^4^ The World Health Organization (WHO) recommends that countries should adopt a methodical approach to coordinated, consistent and competency-based education that is needs-driven and approved. This will ensure that all healthcare workers are equipped with the appropriate competencies to undertake the tasks that they perform.^5^

Competency has been described as the knowledge, perceptions, skills, attitudes and standards that an individual develops or acquires through education, training and work experience, which can be used to depict particular occupational roles or functions against which personal performance may be assessed.^6^ Although it is ideal that all health professionals should be competent to undertake the tasks they perform, competency may be difficult to assess. The assessment of competency in the form of subjective, multiple-choice and standardised patient assessments may underemphasise significant domains of professional capability such as the integration of knowledge and skills, the framework of care, cooperation and patient–provider associations.^7^ It is even more challenging to assess the competency of clinicians in practice. In this study, self-assessment was used to measure how confident nurses are in performing HIV management skills. Knowledge questions were used to provide an objective assessment. Self-assessment is often used to help practising clinicians to identify their own strengths and weaknesses for continuous professional development. However, the process of assessing oneself is complex and never completely objective. Self-assessment can therefore not be used as an accurate measure of competency, but it can be used to help individuals identify gaps in their clinical performance.^8^ One such study conducted in the Eastern Cape province of South Africa found satisfactory self-efficacy in the clinical performance of NIMART amongst 358 trained nurses, with some limitations in their abilities regarding clinical evaluation.^9^ Care provided by providers with low levels of self-rated expertise who treat low numbers of HIV and AIDS patients tends to lead to less favourable patient outcomes.^10^

Factors such as training, mentoring and clinical experience have been found to influence the competency of healthcare providers. A systematic review revealed better clinical outcomes for patients treated by a provider with more training in HIV and AIDS care.^10^ There is very little research available on the evaluation of the different NIMART training courses and training outcomes. One study found that 62% of nurses who had been trained in NIMART were initiating patients on ART in the clinics where they were working, yet some of these nurses did not pass the open book exam after the training.^11^ In KwaZulu-Natal province, knowledge scores of nurses increased significantly after NIMART training, with a median post-test knowledge score of 77%. However, the majority of nurses were not confident enough to practise their skills, which emphasised the need for continuous mentorship.^12^ A study in Khayelitsha, South Africa, showed an increase in the confidence of nurses to manage patients on ART after mentorship.^13^ Similarly, a study evaluating 5 years of NIMART mentorship in South Africa identified improved knowledge, attitudes and confidence perceived by nurses who received NIMART mentoring, but highlighted the need for mentoring to continue in light of continuous changes to treatment guidelines.^14^ In addition to mentoring, provider experience in HIV and AIDS care has shown to improve the quality of care.^10^

Although there is substantial evidence that nurses can provide high-quality treatment and care, several challenges have been identified, such as shortages of essential drugs, salary concerns, excessive workload, lack of practice standards, access to mentoring and infrastructural barriers.^4,11,15,16^ This therefore highlights the need for continuous quality assurance in settings where NIMART is being implemented.^17,18^

No published studies could be found that specifically investigated the factors that influence the knowledge and confidence of nurses currently prescribing ART. Evaluating the HIV management confidence and knowledge of professional nurses who prescribe ART may help to improve ongoing NIMART training interventions. This study therefore aimed to determine the factors that influence the HIV management confidence and knowledge of professional nurses prescribing ART in a rural and urban district in the Western Cape province.

## Methods

### Research design and setting

A quantitative cross-sectional and analytical research design was used because NIMART training has been operational for several years and is in its implementation phase.^19^ The study was conducted in one urban and one rural district – the City of Cape Town (City Health) and the Cape Winelands districts.

### Population and sample

Based on a list obtained from the Department of Health, there were 256 nurses who were authorised to prescribe NIMART in the two districts. Of the five subdistricts in the Cape Winelands, three districts gave permission for the research and two declined. The assessable population, determined by contacting facilities, was 146 (67 in the City of Cape Town and 79 in the Cape Winelands). All the nurses authorised to prescribe NIMART for a period of 1 year were invited to participate in order to account for the clustering effect in the subdistricts. In the Cape Winelands, 49 (69%) participants completed the questionnaires, 18 (25%) refused to participate and four (6%) were absent, on leave or not available when the research was conducted. In the City of Cape Town, 28 (42%) participants completed the questionnaires, 22 (33%) refused to participate and 17 (25%) were absent, on leave or not available when the research was conducted.

### Instrumentation

A self-completion questionnaire was used that was designed by the researcher based on the literature and previous instruments. The questionnaire measured demographic details, influencing factors, HIV management confidence and HIV management knowledge. The questionnaire was available in English only. In addition, facility statistics related to caseload were collected.

### Pilot test

A pilot test was conducted in the Stellenbosch sub-district. Participants in the sub-district were randomly selected to complete the questionnaire. Eight participants completed the questionnaire. After completion of the questionnaire, a few changes were made to the questionnaire. The pilot test data were not included in the main study.

### Validity and reliability

Reliability and content validity of the instrument were ensured by making use of the literature, a review of the instrument by experts in the field of HIV and the pilot test. Content validity was determined by calculating a content validity index (CVI) for each item (called the I-CVI) in the questionnaire based on the feedback of five experts. The I-CVI was calculated by determining the percentage of experts who rated the specific item as relevant. For an item to be considered relevant, four of the five experts had to rate the item as relevant (an I-CVI score of 0.8).^20^ For six items in the questionnaire, one of the five experts rated the item as not relevant (an I-CVI score of 0.8). However, this is still acceptable according to the literature. For the remaining items, all the experts rated the items as relevant (an I-CVI score of 1). They made suggestions to improve the clarity of items and these suggestions were incorporated in the final instrument.

The Cronbach’s alpha coefficient was used to establish the reliability of the Likert scale items that measured confidence. The previously reported Cronbach’s alpha for the confidence items was 0.94.^21^ In this study, the Cronbach’s alpha for the 22 confidence items was 0.95.

### Data analysis

Data were entered into Microsoft Excel by the researcher, imported and analysed using the Statistical Package for Social Sciences (SPSS) software.^22^ HIV management confidence and knowledge were measured as continuous variables by calculating the total scores. The scores were converted to percentages to facilitate interpretation. A higher score indicated more confidence. Descriptive statistics were used to describe the data and appropriate statistical tests were used to test for relationships between variables. As the confidence and knowledge scores were normally distributed, the student *t*-test was used to compare mean scores of two independent groups or categories of influencing factors and analysis of variance (ANOVA) for comparing more than two groups. The Spearman rho correlation coefficient was used to test for associations between continuous variables, for example, scores and the patient caseload of the participant (as the caseload was not normally distributed). A level of significance of < 0.05 was used in this study.

## Ethical consideration

Ethics approval to conduct the study was obtained from the Health Research Ethics Committee at Stellenbosch University (S14/12/268). Permission was further obtained from the Department of Health, City of Cape Town (City Health), Western Cape Province and the appropriate Medical Superintendents from the chosen subdistricts. This study adhered to the ethical principles of the Declaration of Helsinki 2013. The researcher has the ethical responsibility to protect the human rights of the participants, such as their rights to privacy, confidentiality, autonomy, anonymity, fair treatment and protection from discomfort and harm.

## Results

### Biographical data

Most of the participants were recruited from healthcare facilities (*n* = 20, 69%) in the Cape Winelands. The median headcount in facilities was 2496.5 (interquartile range [IQR] 1460.5–5441.5), indicating a high patient load in the facilities with relatively high variability between facilities. The mean age of participants was 43.6 years, with a standard deviation (s.d.) of 9.98 years, the youngest being 25 years of age and the oldest being 64 years of age.

Table 1 presents the categorical biographical data of the participants. Most of the respondents performed clinical work as a professional nurse. Participants in the ‘other’ category were providing different services, for example, the prevention of mother-to-child transmission (PMTCT) manager from head office prescribed ART if there was understaffing in certain clinics. Another participant was appointed as a psychiatric nurse but was also NIMART-trained and provided ART. One participant indicated that she was a clinical mentor but was also allocated to clinics to provide ART.

**TABLE 1:** Biographical data of the participants.

Variable	Frequency (*n*)	%
**Gender**		
Male	6	7.8
Female	71	92.2
**Highest professional qualification**		
Postgraduate diploma in primary healthcare	44	57.1
Diploma	21	27.3
Degree	8	10.4
Other	4	5.2
**Current function**		
Clinical work as a professional nurse	63	81.8
Facility manager	8	10.4
Other	5	6.5
Clinical mentor	1	1.3
**Type of facility**		
PHC clinic	41	53.2
Community day centre	30	39.0
Community health centre	4	5.2
Mobile clinic	1	1.3
Other	1	1.3

PHC, primary healthcare

### Influencing factors

The factors influencing confidence and knowledge included: (1) HIV management experience, (2) training, (3) continuous mentoring and support, (4) workload, motivation, facility equipment and general satisfaction, and (5) quality assurance mechanisms (see Table 2).

**TABLE 2:** Influencing factors.

Influencing factor	Frequency (*n*)	%	Confidence	Knowledge
			*p*	*p*
**1. Experience**				
***Years of experience in HIV management***				
Less than 1 year	4	5.2	0.25	0.45
1 to less than 2 years	25	32.5		
2–5 years	28	36.4		
More than 5 years	20	26		
***Years of experience initiating ART***				
Less than 1 year	6	8.1	0.08	0.16
1 year to < 2 years	34	45.9		
2–5 years	28	37.8		
More than 5 years	6	8.1		
**2. Training**				
***Training course***				
**Time of HIV management training (various courses)** > 3 years 3 years or less	46 31	59.7 40.3	0.06	0.68
**Time of PACK training** > 3 years 3 years or less	47 30	61.0 39.0	0.75	< 0.01
**Time of dispensing course** > 3 years 3 years or less None	32 12 33	41.5 15.5 43.0	0.75	0.01
***NIMART training and mentoring***				
**Time of NIMART training** >3 years 3 years or less	24 53	31.2 68.8	0.14	0.56
**Duration of NIMART mentoring** 1 week 2 weeks > 2 weeks–2 months > 2 months Other	20 5 29 21 2	26 6.5 37.7 27.2 2.6	< 0.01	0.09
**3. Continuous mentoring and support**				
***How often does the ART doctor visit the clinic?*** Daily Weekly Monthly Annually Never	40 28 1 0 8	51.9 36.5 1.3 0 10.4	0.78	0.06
***Are clinical mentors or a supervising clinician for HIV/TB/ART assigned to your clinic or district?*** Yes No	57 20	74.0 26.0	0.49	0.43
***How often do you have contact sessions with your clinical mentor or a supervising clinician?*** Daily Weekly Monthly Annually Never	20 19 14 3 1	35.1 33.3 24.6 5.3 1.8	0.37	0.10
**4. Workload, motivation, facility equipment and general satisfaction**				
***Do you feel your workload is acceptable?*** Yes No	43 34	55.8 44.2	0.65	0.28
***Do you feel motivated towards your work?*** Yes No	68 9	88.3 11.7	0.45	0.34
***Do you feel that the facilities and equipment at the clinic are adequate for the delivery of HIV care?*** Yes No	58 19	75.3 24.7	0.70	0.98
***Are you satisfied with your work conditions (e.g. work environment, salary and work hours)?*** Yes No	40 37	51.9 48.1	0.27	0.31
**5. Quality assurance mechanisms**				
***Feedback is received about***				
**Personal performance relating to prescribing and monitoring patients on ART** Yes No	51 26	66.2 33.8	0.47	0.01
**Performance of the clinic related to the provision of ART** Yes No	62 15	80.5 19.5	0.92	< 0.05

PACK, Practical Approach to Care Kit (flowchart-based ART guidelines);^4^ HIV, human immunodeficiency virus; ART, antiretroviral therapy; NIMART, nurse-initiated and managed antiretroviral therapy.

Note: The median number of monthly ART initiations by participants in the 3 months prior to the study was 7 (IQR 9) and the median number of ART follow-ups was 126.5 (IQR 347).

### Human immunodeficiency virus management confidence

The mean HIV management confidence score was 68.7% (95% CI 66.3–71.1), with a minimum score of 45% and a maximum of 85%. The participants reported the highest confidence in the use of ART stationery (with 51.9% considering themselves experts) and performing a physical examination (with 50.6% considering themselves experts). They were less confident in identifying drug interactions in commonly used medications, because only 14.3% considered themselves to be experts, and stopping or switching drug treatments (with only 15.5% considering themselves as experts). Low confidence was reported in prescribing for concurrent illnesses and in identifying the signs and symptoms of immune reconstitution inflammatory syndrome (fewer than 20% of participants considered themselves experts).

The distribution of the HIV management confidence score was the same across categories of district and facility type. Bivariate analysis indicated a low to moderate positive correlation between the HIV management confidence score and the average number of patients on ART followed up in the last 3 months (*r* = 0.328, *p* = 0.004). The distribution of HIV management confidence scores was not the same across categories of how long NIMART mentoring lasted (*F*[df4] = 4.6; *p* = 0.002). Post hoc analysis revealed that participants who received NIMART mentoring for 2 weeks had significantly higher confidence scores than those who received mentoring for more than 2 months. None of the other influencing factors has significant associations with HIV management confidence (see Table 2).

### Human immunodeficiency virus management knowledge

The mean HIV management knowledge score was 72.7% (95% CI 69.8–75.6). The minimum score was 38% and the maximum 100%. Participants had high knowledge scores regarding treatment for peripheral neuropathy (97.4% indicated the correct answer) and the side effects of tenofovir, with 89.6% indicating the correct answer. High knowledge scores were also identified for identifying pneumocystis pneumonia (88.3% correct) and ART contraindications (88.3% correct). The participants were less knowledgeable regarding the treatment of toxoplasmosis (22.1% correct), oral hairy leukoplakia (39% correct) and tinea capitis (41.6% correct). A few participants (41.6%) demonstrated an understanding of virological failure and only 51.9% provided the correct answer for the question related to drug–drug interactions.

There was a significant difference in distribution of the HIV management knowledge score across categories of district (*t*[df75]= -2.9, *p* = 0.004). Participants in the Cape Winelands had a lower mean knowledge score (69.6%) compared to the mean knowledge score of participants in the City of Cape Town (78.2%). Participants trained in Practical Approach to Care Kit (PACK – flowchart-based guidelines designed for assisting nurses manage various conditions in a primary care setting)^4^ 3 years ago or less had significantly higher knowledge scores (*t*[df70] = -3.5, *p* = 0.001). Those with no training in dispensing had significantly higher knowledge scores, which is an unexpected finding. Knowledge scores of participants who indicated that they received regular feedback about their personal performance (*t*[df75] = 2.45, *p* = 0.016) and the performance of the clinic related to the provision of ART (*t*[df75] = 3.5, *p* = 0.001) were significantly higher compared to those who did not. Bivariate analysis indicated a low to moderate positive correlation between the HIV management knowledge score and the average number of patients initiated on ART in the last 3 months (*r* = 0.357, *p* = 0.002) and a moderate positive correlation for the average number of patients on ART followed up (*r* = 0.386, *p* = 0.001). A significant negative correlation was found between the HIV knowledge score and the average number of other or non-ART patients the participants managed in the past 3 months (*r* = -0.367, *p* = 0.001).

## Discussion

Appropriate training is the beginning of the pathway to expertise.^5,23^ In this study, a variety of HIV management training courses were attended by the participants. All of the participants were trained in PACK, completed an HIV management course and participated in NIMART training as per the NIMART guidelines of the Western Cape.^13^ Only 57.1% of participants had completed a dispensing course, although this did not affect confidence and was associated with lower knowledge scores. A dispensing certificate is not a requirement for NIMART in the Western Cape and most clinics have either a pharmacy assistant or a pharmacist who can dispense medication. Cameron et al.^11^ found that 79% of the nurse participants in their study had previous formal training in HIV management and 55% had formal training in PHC, which is comparable to the 57.1% of participants in the present study who had completed a postgraduate diploma in PHC. A qualification in PHC (Health Assessment, Treatment and Care [R48]) and a dispensing certificate are therefore not requirements to prescribe ART in the study context, but the PACK training and completing an HIV management course are. Although no cause and effect can be inferred, it can be deduced from the results that recent PACK training (3 years or less) is likely to improve the HIV management knowledge of nurses.

Clinical mentoring is depicted alongside clinical practise and continuous assessment on the pathway to competency and proficiency.^22^ The purpose of mentoring is to acquire skills to competently initiate, but also manage patients according to established clinical protocols.^17^ The Western Cape Department of Health guideline advises a minimum of 40 h of NIMART one-on-one mentorship following didactic training.^13^ In a study in the health districts of Tshwane (Gauteng Province), Nkangala (Mpumalanga Province) and Capricorn and Vhembe (Limpopo Province), the median mentoring period was 25 months.^14^ It therefore appears that there is no set period for mentoring. A total of 80 cases are required to be seen by a nurse in consultation with a mentor, according to the Clinical Mentorship Guideline for Integrated Services, in order for the nurse to be authorised in NIMART.^22^ In the present study, all nurses had received NIMART mentoring. The findings from this study support a 2-week NIMART mentoring period. It may be that a 2-week mentoring period is more intense. Perhaps those who were mentored for a longer period, for example, for more than 2 months, did not have intensive contact sessions and case studies or the contact sessions may have been too far apart. A study conducted by Orner et al.^16^ found that nurses and doctors were too busy for mentoring, which may result in less frequent contact sessions. A longer period of mentoring may therefore not translate to the acquisition of more knowledge and confidence in practice. This, however, needs to be explored further. Having an assigned mentor or the frequency of contact sessions with the mentor were not associated with the participants’ level of confidence or knowledge. However, other studies have found mentoring to improve nurses’ confidence, improving institutional barriers and the quality of patient care.^13,14^

Although mentoring was not associated with the participants’ knowledge in the present study, participants who received regular feedback about their personal and clinical performance (classified in this study as part of quality assurance) had significantly higher knowledge scores (75.2%) compared to those with limited or no feedback (67.9%). Feedback was mostly received from the clinic manager or HIV and AIDS, STIs and TB coordinator. Regular feedback provided about individual and clinic performance related to the provision of ART is likely to influence nurses’ HIV management confidence.^4^ However, the Clinical Mentorship Manual for Integrated Services^22^ distinguishes between clinical mentorship and supportive supervision. Although both have similar goals and some overlapping activities, supervision tends to emphasise health facility management and is more hierarchical, whereas mentoring is more focused on the enhancement of the skills of the mentee. Mentoring should therefore be more effective in improving the confidence and knowledge of nurses than feedback from supervisors. The above mentioned results may mean that mentoring is not currently being implemented or practised effectively.

Knowledge and confidence of the clinicians should increase as they gain experience.^22^ In this study, the HIV management confidence and knowledge scores were the same across all categories of experience. However, the average number of patients living with HIV seen or the caseload may be an indication of the intensity of experience and was associated with both HIV management confidence and knowledge. It therefore appears that the total years of experience does not influence confidence and knowledge, rather it is more clinical practice experience, for example, the patients living with HIV caseload, that influences confidence and knowledge. None of the other health system influencing factors were associated with HIV management confidence and knowledge. These may nonetheless still have an impact on overall service delivery and quality of patient care.

Recommendations based on the study findings include regular guideline updates to reinforce HIV management knowledge. Methods to strengthen mentoring should be engaged such as dedicated roving mentors or access to telephonic consultations.^14^ Dedicated mentoring may be costly and therefore non-governmental organisation support may be required to ensure mentor capacity.^13^ NIMART-trained nurses should be offered sufficient opportunities to practise their skills. As not all nurses may be exposed to a high caseload because of the PHC approach of providing integrated services, especially in rural settings, those with low caseloads need access to updated guidelines and mentors to assist them when they initiate patients or encounter complex cases.

### Limitations

Study limitations include the small sample size and high refusal rate, which limits the generalisability of the findings. The small sample size further limits the power of the statistical tests to detect significant differences. As mentioned before, self-assessment may not accurately measure confidence. The cross-sectional nature of the study implies that no cause and effect relationships between the variables explored can be inferred. Further, the knowledge and competency items assessed were delimited to adult HIV care.

## Conclusion

This study investigated various factors that influence the knowledge and confidence of professional nurses prescribing ART in an urban and rural setting, and gave an account of what is currently happening at the healthcare facilities that partook in the study. The majority of participants had adequate HIV management knowledge and reported being very confident or experts in the HIV management skills and competencies. Training, mentorship and clinical practice experience are associated with knowledge and confidence. Recommendations include the strengthening of current training and mentoring programmes and ensuring that NIMART-trained nurses are provided with regular updates and sufficient opportunities for clinical practice.
